# Mitigating the Impact of Emerging Animal Infectious Disease Threats: First Emerging Animal Infectious Diseases Conference (EAIDC) Report

**DOI:** 10.3390/v14050947

**Published:** 2022-04-30

**Authors:** Suresh V. Kuchipudi, Deanna Behring, Ruth Nissly, Shubhada K. Chothe, Abhinay Gontu, Abirami Ravichandran, Ty Butler

**Affiliations:** 1 Animal Diagnostic Laboratory, Department of Veterinary and Biomedical Sciences, The Pennsylvania State University, University Park, PA 16802, USA; rah38@psu.edu (R.N.); skc172@psu.edu (S.K.C.); abhinay@psu.edu (A.G.); aur1121@psu.edu (A.R.); 2 The Center for Infectious Disease Dynamics, Huck Institutes of the Life Sciences, The Pennsylvania State University, University Park, PA 16802, USA; 3 College of Agricultural Sciences, The Pennsylvania State University, University Park, PA 16802, USA; dbehring@psu.edu (D.B.); iyb5026@psu.edu (T.B.)

**Keywords:** EAIDC, emerging infectious diseases, avian influenza, foot and mouth disease, African swine fever

## Abstract

From 29 November to 1 December 2021, an “emerging animal infectious disease conference (EAIDC)” was held at the Pennsylvania State University. This conference brought together distinguished thought leaders in animal health, veterinary diagnostics, epidemiology and disease surveillance, and agricultural economics. The conference’s primary objective was to review the lessons learned from past experiences in dealing with high-consequence animal infectious diseases to inform an action plan to prepare for future epizootics and panzootics. Invited speakers and panel members comprised world-leading experts in animal infectious diseases from federal state agencies, academia, professional societies, and the private sector. The conference concluded that the biosecurity of livestock operations is critical for minimizing the devastating impact of emerging animal infectious diseases. The panel also highlighted the need to develop and benchmark cutting-edge diagnostics for rapidly detecting pathogens in clinical samples and the environment. Developing next-generation pathogen agnostic diagnostics will help detect variants of known pathogens and unknown novel pathogens. The conference also highlighted the importance of the One Health approach in dealing with emerging animal and human infectious diseases. The recommendations of the conference may be used to inform policy discussions focused on developing strategies for monitoring and preventing emerging infectious disease threats to the livestock industry.

## 1. Introduction

Emerging infectious diseases (EID) are recognized as pathogens whose incidence in host populations has increased within the past two decades or threatens to increase in the near future. African swine fever (ASF), which recently re-emerged in China and subsequently spread to several countries, is associated with high morbidity and mortality. Its high stability and ability to quickly spread, as well as the lack of effective vaccines, make the ASF virus (ASFV) a severe threat to the global swine industry and national economies [1]. Highly pathogenic avian influenza (HPAI) H5 viruses of the A/goose/Guangdong/1/1996 (Gs/GD) lineage continue to evolve into diverse clades and cause outbreaks in countries worldwide [2]. Since 2014, H5Nx HPAI viruses belonging to clade 2.3.4.4., including H5N2, H5N6, and H5N8, are circulating globally in wild birds and poultry, causing substantial economic losses for the poultry industry [3]. HPAI H5N1 viruses are zoonotic; they transmit from infected poultry to humans, often with fatal consequences [4], It is worth noting that between 60 and 80% of newly emerging infections are zoonotic in origin [5].

The College of Agricultural Sciences convened an emerging animal infectious disease conference (EAIDC) at Pennsylvania State University in partnership with the Pennsylvania Department of Agriculture and the Penn State Center for Security Research and Education (CSRE) between 29 November and 1 December 2021. EAIDC brought the relevant stakeholders together to learn from past experiences in dealing with high-consequence animal infectious diseases and prepare for future epizootics and panzootics. EAIDC included four scientific sessions followed by breakout sessions. In Session 1, world-leading experts gave an overview of the significant ongoing threats to the animal industry. Session 2 focused on the lessons learned from the previous infectious disease epizootics. In Session 3, speakers addressed the economic implications of animal infectious diseases. In Session 4, experts discussed the advances and future opportunities in our ability to monitor, prevent, and control disease outbreaks.

EAIDC also included three parallel breakout sessions, each focusing on one major animal industry (cattle, swine, and poultry) to discuss and develop a list of critical challenges and potential solutions. The breakout sessions were attended by stakeholders from industry, academia, and regulatory agencies, and the topics discussed included biosecurity, disease modeling and forecasting, vaccines, diagnostics, regulatory issues, and economics.

## 2. Convening Remarks

Jenny Lester Moffit, the United States Department of Agriculture Under Secretary for Marketing and Regulatory Programs, who delivered the Keynote address, emphasized the critical nature of research in sustainable food supply and food security, particularly in animal agriculture, since animal and human health are interconnected. She highlighted that local, national, and international partnerships for sustaining animal agriculture industries are critical because of the large economic impact of food animals and outlined USDA efforts to prepare for and combat highly pathogenic avian influenza, virulent Newcastle disease, African swine fever, and foot and mouth disease. Under Secretary Moffit concluded by announcing that new USDA funding is becoming available for animal disease preparedness. Pennsylvania Department of Agriculture Secretary Russell Redding noted the importance of combatting animal disease in the state of Pennsylvania, where 340,000 jobs are supported by an animal agriculture industry with more than 200 million animals.

## 3. Scientific Presentations

### 3.1. Session 1: Threats to the Animal Industry

Dr. Peter Fernández, a consultant at the PJF AgroStrategies Consulting, presented his talk on “Animal Disease Threats–Past, Present, and Future”. He discussed the threats animal diseases pose to humans in terms of zoonotic spread, food security, and trade and economy. He used the examples of past and present animal diseases to explain various lessons from these experiences. Using historical learnings from the past animal disease threats Rinderpest and contagious bovine pleuropneumonia (CBPP), Dr. Fernández made a case that it is possible to eradicate diseases globally through coordinated international efforts and, in the process, develop novel technology, strategies, and infrastructure. He also observed that successful eradication would involve intensive surveillance, effective and transparent tracking of disease, and investment and that simple interventions for control and eradication can be effective. Using the present disease foot and mouth disease (FMD), Dr. Fernández stated that control programs, such as trade restrictions, need to evolve to match risk pathways. Using the example of the present disease African swine fever he stated that disease threats can have a wildlife origin, and the introduction of these animals into endemically stable environments can lead to disease. He quoted the example of Bovine Spongiform Encephalopathy to state that some diseases may have human-induced facilitated transmission. From West Nile virus, he stated that the development of effective vaccines can minimize the impact of disease threats. To control future challenges, Dr. Fernández stated that continual enhancement of diagnostic technologies; development of therapeutics, such as vaccines and antimicrobials; coordinated surveillance programs; and emergency response systems at federal, state, and local levels are necessary. He concluded by stating that understanding complex factors that lead to disease emergence, improving data collection, and empowering communities to proactively monitor and respond to diseases would be the way forward to address animal disease threats.

Dr. Ian Brown, head of virology at the Animal and Plant Health Agency, Weybridge, and director of OIE/FAO International Reference Laboratories for Avian Influenza, Newcastle Disease, and Swine Influenza, gave a presentation titled “Repeated Threat to Europe from HPAI-Science Informing Lessons Learned”. In this presentation, Dr. Brown discussed the intercontinental spread of the HPAI H5N8 viruses and highlighted the role of wild birds, sentinel animals, and bridge species in mediating the spread and evolution of the H5Nx viruses. Dr. Brown further discussed the currently dominant H5 clade 2.3.4.4 and its evolution and epidemiology in the European region. He stated that the HPAI H5N8 outbreak of 2020–2021 in the UK has been the largest-ever avian influenza outbreak in the UK and highlighted that biosecurity is the key in dealing with such situations. Dr. Brown further reviewed the immediate and future challenges that the poultry industry may face in the prevention and control of infectious agents, such as influenza viruses. These challenges included maintaining a high biosecurity/hygiene standard as part of normal practice and constantly developing antigenically matched influenza vaccines to the field strains. He concluded the talk by emphasizing that, while the zoonotic danger is considered ‘low’, a continuous assessment of the zoonotic risk involved is essential.

Dr. Donald King, head of the Vesicular Disease Reference Laboratory Group at the Pirbright Institute, presented a talk titled “Foot-and-Mouth Disease (FMD): A positive perspective on global disease surveillance and control initiatives achieved via international partnerships”. FMD is a disease of cloven-hoofed livestock and wild animals. It is estimated that FMD causes an annual economic loss of around USD 6.5–21 billion worldwide. Dr. King discussed the dynamic epidemiology of FMD by dividing the endemic geographical regions into seven major pools that host various combinations of the seven FMD serotypes. He further highlighted the possible underlying causes of FMD movement across these endemic regions as animal movement; community migration or migration of animal products; and newly expanding opportunities, such as road building, etc. One such example is the expansion of the FMS lineage O/ME-SA/Ind-2001, which has had multiple ‘escapes’ since 2013 in the South Asian region. Dr. King further reviewed the potential gaps that hinder the global FMD control, which include nonimmune and intensively reared livestock, fewer incentives for FMD control in the endemic regions, and the lack of appropriate resources to combat the burden of FMD. He concluded the talk by emphasizing the importance of improving disease surveillance in countries where veterinary resources are limited and using this data from different sources to develop predictive models of FMD spread.

### 3.2. Session 2: Infectious Disease Epizootics—Lessons Learned

Dr. Falko Steinbach leads the Mammalian Virology Group at the United Kingdom’s Animal and Plant Health Agency and is a professor of Veterinary Immunology at the University of Surrey. The central theme of the presentation was that no single solution can control disease. Each of the viral diseases presented responded differently to current vaccines. A differentiating infected from vaccinated animals (DIVA) vaccine against Suid herpesvirus 1 (the cause of Aujeszky’s disease) provided reduced disease of infected animals but did not prevent transmission. On the other hand, a highly effective vaccine against classical swine fever virus stopped transmission but did not hold DIVA properties, thus complicating trade. In the case of the alphacoronavirus porcine epidemic diarrhea virus, vaccines that protect piglets at a very young age are needed to control the disease and prevent recombination with other coronaviruses. The wild animal reservoirs of porcine viruses are a further consideration for controlling viral diseases in that administering vaccines to such populations is most effective via oral vaccine-laced baits, which are not live vaccines, largely precluding DIVA approaches.

Dr. Fidel Hegngi, a senior staff veterinarian at the United States Department of Agriculture Animal and Plant Health Inspection Service Veterinary Services, provided insight into the “Stamping Out” approach to controlling highly pathogenic avian influenza virus using examples from previous outbreaks in the US. Dr. Hegngi summarized that effectively stamping out avian influenza by depopulation and quarantine instead of through treatment efforts was most effective through a unified but locally driven response. Utilizing local resources and establishing mentoring relationships between experienced farmers and newly affected farmers helped to encourage adherence to biosecurity, quarantine, and containment protocols to minimize disease impacts. Interestingly, the mechanism of disease spread in historical (1924) and recent (2014–2015) outbreaks was the same: vehicles used for transportation between poultry-inhabited premises. Dr. Hegngi highlighted the role of humans in avian influenza virus control, not the least of which is the requirement of human manpower for outbreak response and the subsequent need to keep these individuals safe in the process. Establishing response teams during non-outbreak times allows these teams to form, develop, and practice to enable a successful response whenever “game time” arrives.

### 3.3. Session 3: Infectious Disease–-Economic Implications

Dr. Alan Olmstead, distinguished research professor of economics at the University of California at Davis, provided an exciting historical perspective of government institutions’ roles in controlling and eradicating animal diseases in the United States. His talk followed the story of the US Bureau of Animal Industry (BAI). While the BAI was able to eradicate contagious bovine pleuropneumonia in the 1880s–1990s using its independent powers, once it was able to enforce interstate commerce and federal quarantines through cooperative agreements, disease eradication was much more rapid. The work of the BAI in controlling disease in the US was difficult and faced much opposition, but the education of lawmakers and judges helped to enable success. In addition to BAI’s health enforcement roles, it also was the research institution that established the first US government biological laboratory. In this laboratory, breakthroughs in understanding tick-borne disease transmission; the discovery of bacterial, viral, and parasite disease agents; and the creation of the US’s first heat-killed vaccine took place. Among the many lessons to be drawn from the BAI, one particularly stands out: in the face of an enormous disease problem, start eradication efforts in areas in which their success is most likely, as success breeds success.

Using African swine fever as an example, Dr. Kevin Grier of Kevin Grier Market Analysis and Consulting, Inc., explained how an infectious disease outbreak could lead to structural changes in not only the affected livestock industry but also multiple inter-connected agricultural industries. Disease-related export bans are projected to cause a pork price collapse. This drop would force the liquidation of herds and assets, and the altered prices could alter consumer preferences. Furthermore, the producer and consumer shifts caused by an extended period of export ban would lead to a complete structural change in the food animal industry. Outbreak exercises offer an opportunity to forge working relationships between stakeholders during non-emergency periods, improving responses to minimize the economic impacts of a potential food animal disease.

Dr. Dustin Pendell, Professor of Agricultural Economics at Kansas State University, described some of the complications of the economics of outbreaks. While producers and the agricultural industry in general suffer huge losses, responses to outbreaks typically provide a temporary economic boost to local economies. The demand for hotel rooms, restaurant workers, and other supporting entities increases with the arrival of disinfection crews and outbreak investigators. Direct losses to the animal industry can be significantly reduced with government interventions, such as conducting depopulation and establishing quarantine areas. In addition to government-funded response and indemnities, animal industry producers can reduce losses through the proactive risk assessment of their company and the development of business continuity plans, modeled by the Secure Egg Supply Program. Finally, the economic models of US African swine fever outbreaks and the actual results of the 2014–2015 US outbreak of highly pathogenic avian influenza demonstrate that an economic impact on one food animal sector has direct consequences on commodity prices in all other agricultural sectors. Planning exercises simulating an animal disease outbreak can bring together the key players for disease response during non-emergency times, forging important team relationships.

### 3.4. Session 4: Monitoring, Prevention, and Controlling Outbreaks

Dr. Robin Holland, head of the Diagnostic Services Section, Foreign Animal Disease Diagnostic Laboratory (FADDL), USDA, presented an overview of the current and future priorities of the USDA. Dr. Holland described the current role of the National Veterinary Services Laboratories (NVSL) in safeguarding the livestock of the country. Dr. Holland highlighted that foot and mouth disease (FMD), African swine fever (ASF), and classical swine fever (CSF) are currently on the priority list for USDA activities. In the future, this list is being expanded to include diseases like Nipah, Japanese encephalitis, Rift Valley fever, and Crimean Congo hemorrhagic fever. Dr. Holland concluded by introducing the establishment of the National Bio and Agro-defense Facility (NBAF), which will be a state-of-the-art biocontainment laboratory for the study of diseases that threaten both the US animal industry and public health.

Dr. Juergen A. Richt, Regents distinguished professor, College of Veterinary Medicine, Kansas State University, presented a talk entitled “African Swine Fever (ASF): Development of Vaccines and Point of Need Diagnostics”. Dr. Richt elucidated the currently available vaccination strategies for African swine fever. He discussed the results from safety and efficacy studies of modified live virus vaccines and advocated their use for preventing ASF. Later, Dr. Richt described molecular approaches for the diagnosis of ASF. He finished by emphasizing the need for rapid and point-of-need diagnostics for the effective control of the disease.

Dr. John M. Hardham, director of Zoetis Center for Transboundary and Emerging Diseases, provided an overview of the challenges and opportunities caused by the emerging infectious disease from an animal health perspective. In his talk, Dr. Hardham introduced new vaccines under development for FMD, ASF, and CSF. Later, Dr. Hardham described the COVID-19 infections in animals. He presented study results of a recombinant spike protein-based vaccine for COVID-19 for veterinary use at Zoetis. Dr. Hardham ended the presentation with the vaccine’s ability to induce serological responses that can cross-neutralize the variants of severe acute respiratory syndrome coronavirus 2 (SARS-CoV-2).

### 3.5. Session 5: Animal Industry Challenges

Dr. Brigitte Mason, a senior veterinarian at Country View Family Farms, discussed the largest challenges in the swine industry: labor, disease, and new regulatory requirements. The quickly growing swine industry faces challenges in hiring and retention of employees. The rural labor markets where most swine production occurs have been significantly reduced during the industry growth. While immigrant workers have minimized the loss of native labor and enabled the continuity of the rural economies, a labor shortage remains because of visa costs and limitations on the duration of agriculture work visas. The major disease challenges are porcine reproductive and respiratory syndrome, *Mycoplasma hyopneumoniae*, *Streptococcus suis*, hemolytic *E. coli* F-18, ASF, classical swine fever, and FMD. Producers are encouraged to develop a Secure Pork Supply Plan for business continuity, and multiple organizations are available to help planners with this task. Finally, Dr. Mason described new regulations aimed at improving animal welfare and worker safety that force producers to make new calculations about space allocations, herd size, and production volume to remain compliant.

Dr. Kevin Brightbill, the state veterinarian for Pennsylvania, presented the most urgent concerns facing the cattle industry. The state of Pennsylvania’s cattle industry is predominated by small dairies and cow/calf farms, which must compete on a global market with large producers and cheaper imports while managing limitations of new farmland availability, changing weather patterns, and contracting the next generation of farmers and large animal veterinarians. Biosecurity remains an enormous challenge to the cattle industry since the nature of large animal production and family farming involves different access restriction abilities compared with the poultry and swine industries. In the context of disease threats, bovine respiratory disease remains the leading cause of morbidity and mortality in cattle. Insect-transmitted diseases are emerging as a novel threat in Pennsylvania because the range of *Aedes* mosquitoes and Asian longhorn ticks is increasing. The threat of foot and mouth disease has made the ability to trace animals and disease more urgent, so the increased use of radio-frequency identification tags on individual animals and the support of disease detection are important.

Dr. Eric Gingerich, a poultry technical specialist at Diamond V, presented the greatest challenges to the poultry industry. Biosecurity and preparedness to prevent and respond to viral diseases, such as avian influenza, are key challenges. High employee turnover has created a small workforce trained to respond to disease outbreaks. Supplies for whole-house euthanasia, depopulation, and disposal as well as more efficient and cost-effective approaches, such as ventilation shutdown, are needed. Egg drop syndrome in brown egg layers is another viral disease significantly pressuring the industry with no approved vaccines in the US. Further research on infectious sources, detection, and vaccines is urgently needed. The control of this disease would highly benefit from permission to utilize vaccines that are approved in other countries. A summary of the research priorities identified by the American Association of Avian Pathologists was provided. These priorities included autogenous vaccine approaches; vaccines against reovirus, infectious bronchitis, and infectious coryza; non-antibiotic control of colibacillosis, coccidiosis, and histomoniasis; and the improved understanding and detection of avian infectious diseases.

## 4. Discussion Groups on Animal Sectors

### 4.1. Swine

Biosecurity needs within the swine industry are focused on better outreach to both small feed mills and off-species feed mills regarding biosecurity best practices, which also require the construction of uniform, industry-specific biosecurity standards. Also encouraged was the promotion of a voluntary program to avoid taking feed from “high-risk” feed mills, although data collection is needed to set definitional standards and work directly with feed producers. There is an additional concern that as diagnostic labs advance technologically, the skills and resources of the diagnosticians themselves may not keep pace, leading to lower quality data interpretation. Samples submitted for review should be encouraged to have strong animal and herd histories to better allow diagnosticians to interpret the data that they find. Relationship-building between farmers and diagnosticians would further this goal as well. In regulatory terms, screening models for feed (such as those in place in Canada) are often deemed “too expensive” to implement through formal regulations in domestic markets, and optional buy-in should be promoted. This, however, will depend on the availability of regularly updated, high-quality feed source data. Other efforts, such as storage treatment methods and third-party source monitoring, could reduce risk as well. Key gaps exist, however, in research on organic vs. non-organic differences and best practices for adequately testing larger and varied containers of feed. In terms of labor availability, year-round H2A visas would help address acute labor shortages within the industry. Additional funding to implement apprenticeship models, particularly through 4H programs, could help with long-term labor availability from domestic sources as well.

### 4.2. Cattle

With the decline in regional cattle farm numbers, major concerns have arisen about the ability of local farms to compete in an increasingly global marketplace. The northward incursion of new insect-borne diseases and changing weather patterns, leading to increases in both droughts and flooding, have impacted cattle feed production, encouraging selloffs of cattle prior to winter months at reduced markets rates. Climate change has brought with it new disease vectors. Large animal veterinarian availability is currently limited, reducing access for cattle health needs. This is particularly concerning when coupled with the general lack of both updated biosecurity standards and the trained personnel needed to combat a potential mass disease outbreak (such as foot and mouth disease). Farm access and worker health screening need to be more closely monitored and encouraged to prevent contamination. RFID tagging and a crack-down on animal ID violations at local markets can help to better track and contain infected animals in the case of an outbreak.

### 4.3. Poultry

The discussion group on the poultry sector highlighted the importance of developing new incentives for poultry producers and companies to do more to enhance biosecurity. This is particularly important for the live bird market, and support for the USDA Live Bird Market working group was articulated. Novel suggestions for incentives included working with agriculture lenders to require biosecurity plans or proof of certified poultry technician training as part of loan applications. More research and public education about the economic losses related to outbreaks could also serve to incentivize action. While we have a good lab system in the United States, we need to continue to invest in staff and facilities, and the shortage of poultry veterinarians needs to be attenuated. Regarding mitigation efforts, testing is an important area of focus, particularly among the wild bird population, where the risk is greatest. The management of the euthanasia process is important, as is training and research into new methods. In particular, we need better methods for large, commercial flocks. Shut-down and disposal training are also important, as these actions can make an enormous difference in how an outbreak plays out. Finally, better communication and advocacy with vaccine companies need to be coordinated, particularly for diseases that currently depend on autogenous vaccines and overlap with the broiler and layer industries (such as bronchitis and reovirus).

## 5. Concluding Comments and Recommendations

Concluding comments included a summary by the secretary of agriculture for Pennsylvania, Russell Redding, who impressed upon attendees the need for action. COVID-19 has afforded our community an opportunity by bringing to light to the public the critical role that the animal agriculture industry plays in our welfare, as it was designated a “life-sustaining” industry during the pandemic. Secretary Redding also urged a focus on preparedness rather than response, as the reaction to crises is to implement restrictive measures, which COVID-19 has shown to be problematic. Redding also acknowledged the importance of considering both the “science” and the “civics” of the problem and that the best preparedness will include the consideration of social behaviors and depend upon collaborations among people from around the world. 

Dr. Suresh Kuchipudi then summarized the key take-away messages of the conference. First, emerging animal infectious diseases are not just a health threat but also affect our agriculture economy and ancillary industries, such as hospitality and construction. Therefore, preparedness is critical, and we should consider lessons learned from past outbreaks to help us with strategies for the future. A shared understanding of biosecurity is vital to translate the written policies into tangible and practical actions. Understanding the risk factors for disease transmission at the animal–human interface may identify disease prevention and outbreak containment opportunities [6].

There is a need to develop and benchmark diagnostics that can detect pathogens in clinical samples and the environment. Investing in the development of next-generation pathogen agnostic diagnostics will help detect variants of known pathogens and unknown novel pathogens. Pathogens continue to evolve, and genetic signatures in pathogen phylogenies allow us to look back at the underlying ecological selection pressures and the emergence of novel pathogens [7]. 

The conference also highlighted the importance of the One Health approach in dealing with emerging animal and human infectious diseases. The concept of One Health aims to drive improvements in human, animal, and ecological health through a holistic approach [8]. The One Health approach to tackling zoonotic diseases considers all components that might lead to or increase their threat, including environmental, ecological/wildlife, domestic animal, and human factors [9]. The conference ended with an appreciation for the speakers, participants, and planners and their efforts to take a truly comprehensive view of the emerging animal infectious disease threat. 
